# Efficacy of a joint didactic intervention using the Junta De Andalucía School for Patients method to control prothrombin time in patients taking anticoagulants: protocol for a randomized controlled trial

**DOI:** 10.1186/s13063-020-04972-1

**Published:** 2021-01-11

**Authors:** Leovigildo Ginel-Mendoza, Alfonso Hidalgo-Natera, Rocío Reina-Gonzalez, Rafael Poyato-Ramos, Juana Morales-Naranjo, Inmaculada Lupiañez-Pérez, Antonio Baca-Osorio, Miguel Gutiérrez-Jansen, María Paz Fernández-Lara, Diego Lozano-Noriega, Ulises Salgado-Carvallo, Cristina Bandera-García, Francisco Javier Navarro-Moya

**Affiliations:** 1 Centro de Salud Ciudad Jardín, Distrito Sanitario Málaga-Guadalhorce, C/ Sancho Miranda 9, 29014 Málaga, Spain; 2 Andalusian Health Service, District of Málaga-Guadalhorce, Málaga, Spain

**Keywords:** Anticoagulants, acenocoumarol, warfarin, health education, Patient participation, Conditions: health education, patient education as topic, and blood coagulation, Randomized control trial

## Abstract

**Background:**

Oral anticoagulant drugs represent an essential tool in the prevention of thromboembolic events. The ones in widespread use are vitamin K antagonists, whose plasma level is monitored by measuring prothrombin time using the international normalized ratio. If its values are out of the recommended range, the patient will have a higher risk of suffering from thromboembolic or hemorrhagic complications. Previous research has shown that approximately 33% of patients keep having values at an inappropriate level. The purpose of the proposed study is to improve the international normalized ratio control results by a joint didactic intervention based on the Junta de Andalucía School for Patients method that will be implemented by anticoagulated patients themselves.

**Methods:**

A randomized controlled trial will be undertaken at primary care centers from one healthcare area in Málaga (Andalusia, Spain). Study population: patients participating in an oral anticoagulant therapy program of vitamin K antagonists.

First step: identification of patients in the oral anticoagulation therapy program with international normalized ratio control of the therapeutic level at 65% or less over total time. Second step: patients with international normalized ratio (INR) control figures under 2 or above 3 will be assigned to two different groups:

Group 1 or joint intervention group: patients will be instructed in the joint didactic “from peer to peer,” by a previously trained and expert anticoagulant patient.

Group 2 or control group: the control group will receive the usual clinical practice. They will be evaluated by nurses about once a month, except for cases in which their INR figures are under 2 or above 3, and those patients will be evaluated more frequently.

A total of 312 individuals will be required (156 in each group) to detect differences in INR figures equal to or higher than 15% between the groups.

Study variables: time on therapeutic levels before and after the intervention; sociodemographic variables; vital signs; the existence of cardiovascular risk factors or accompanying diseases in the clinical records; laboratory test including complete blood counts, bleeding time, and prothrombin time or partial thromboplastin time; and blood chemistry, other prescribed drugs, and social support.

A quasi-experimental analytic study with before-after statistical analysis of the intervention will be conducted. Linear regression models will be applied for the main variable results (international normalized ratio value, time on therapeutic level) inputting sociodemographic variables, accompanying diseases, and social support.

**Trial registration:**

ClinicalTrials.gov NCT03647254. Registered on 27 August 2018

## Background and current status

It has been proven that oral anticoagulant therapy (AOT) is effective in preventing situations with a high risk of thromboembolic events and in treating them [1, 2]. It significantly decreases ischemic stroke rates as well as their severity and associated death risk.

However, it is a therapy with special features, such as doses and response variability, a narrow therapeutic range, interactions with other drugs and foods, and potentially severe side effects. These facts compel the investigators to undertake close monitoring of these patients [2].

An important effect in developing cardioembolic or hemorrhagic events due to inappropriate coagulation control values using vitamin K antagonists (VKA) has been found, because control variations around 15% could result in relevant clinical problems [1].

The main AOT indications are deep vein thrombosis, pulmonary embolism, and embolic condition prevention in patients suffering from atrial fibrillation or heart prosthetic valve wearers [3, 4]. Atrial fibrillation (AF) is currently the pathology that has the most anticoagulant therapy indications. This fact is partially due to the aging of the population, since there are both incidence and prevalence increases of this arrhythmia, among older patients [5, 6]. Control of patients on AOT was performed in hospitals until a few years ago. Nevertheless, changes in social and health contexts besides the expansion of the use of these drugs have placed them in the purview of primary health care. The development of handheld coagulation measuring devices and the need to improve patients’ accessibility had been used to justify the decentralization of control. Currently, AOT monitoring is carried out on stable patients in primary care [7, 8].

When a need for receiving anticoagulation has been established, this could be implemented by using heparin or vitamin K antagonists (acenocoumarol or coumadin) or newer direct action oral anticoagulants such as dabigatran, rivaroxaban, apixaban, or edoxaban [9, 10].

A method of standardizing prothrombin time results, called the international normalized ratio (INR) system, allows for reliable results to be obtained from different laboratories [11].

The development of coagulation handheld measuring devices to match INR in the capillary blood with a high sensitivity and reliability has made anticoagulant therapy controls in primary care possible. Their use allows for a comprehensive approach and control of these patients.

The number of patients with AF on anticoagulation with VKA is high; however, the INR level control results are not ideal [12–14].

Through educational and behavioral interventions, VKA-anticoagulated patients can increase their knowledge and understanding of the action mechanisms of anticoagulant drugs. As a consequence, patients may increase their ability to maintain INR appropriate control. A recent study regarding these interventions noted that education is particularly important to provide information about safety for patients suffering AF on AOT. In addition, education allows them to make their own well-founded decisions about treatment options and to manage their AOT. However, there are not enough evidences to establish definite conclusions about these interventions [15]. However, three research articles recently conducted in Spain show high out of range anticoagulation control levels in patients on VKA [16–18].

However, there is not enough evidence to establish definitive conclusions about educational health interventions on the INR behavior of patients suffering from AF on OAT [19, 20].

Furthermore, it should be noted that these chronic patients are prone to suffer from many other conditions and, as a result, they are likely to take many medications. This factor makes health self-management less effective. Hence, educative aspects aimed at self-care and effective health self-management are necessary components of educational interventions targeting this type of patients [21, 22]. Self-care support programs are presented as a tool to accomplish a change. This tool or method is also presented as an alternative to the paternalistic method in which citizens could receive more information (this fact is in accordance with a demonstrated increase in people’s request for health issues via the Internet).

### Aim

The present study aims to show that conducting an organized group educational intervention on VKA-anticoagulated patients by using the Junta de Andalucía School for Patients method will result in reductions in inappropriate INR control levels.

## Methods

The investigators will perform a randomized single-blind trial over a period of 28 months. The study started on 1 September 2018 and will end on 31 December 2020.

### Study area

One healthcare area in Málaga (Spain) where 741,000 inhabitants from rural or urban population are cared for. It is organized into 33 primary care centers and 19 local doctors’ offices.

### Study population

Participants are affiliated to four different primary care centers representing people from different social classes in Málaga city.

### Inclusion and exclusion criteria

#### Inclusion criteria

- Men and women over 18 years old affiliated to one of the 4 primary care centers selected
- Patients on VKA treatment for at least the last 6 months in a primary care environment
- Patients showing INR level control values under 2 or above 3 (out of range)
- Patients for whom we have access to, at least, 80% of their INR level controls in the last 6 months of treatment with VKA, even though they are enrolled in another primary care center
- Patients who give written informed consent to take part in the study

#### Exclusion criteria

- Patients suffering from cognitive impairment that prevents them from understanding what is written in the information sheet and consent form
- Limited mobility patients
- Terminal patients
- Alcoholism or drug addiction
- Severe psychiatric illness
- Any other reason that prevents them to be present at center’s meetings

### Setting the limits and justification of the sample size

The total population from the health centers participating in the study amounted to approximately 50,000 inhabitants.

#### Step I

**Fig. 1:**
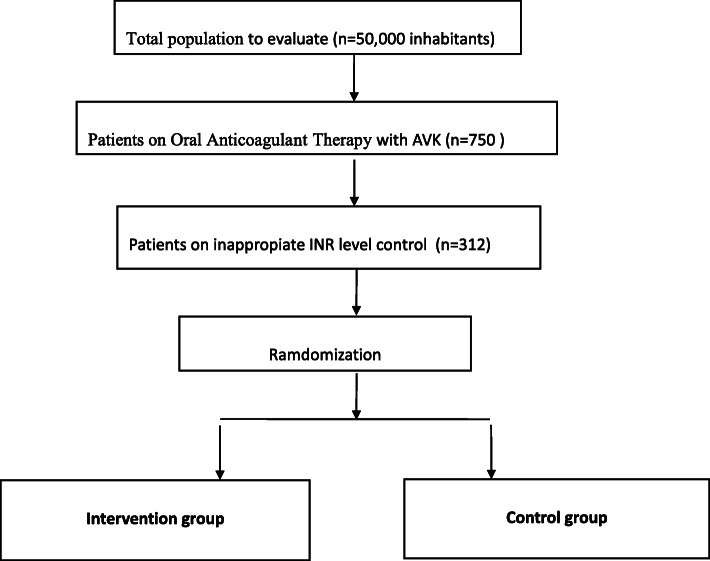
Study flow chart

Considering that the prevalence of patients on AOT is about 1.5% of the general population, it can be claimed that the target population to initiate our study will be 750 anticoagulated patients (using anticoagulant drug consumption data from our health área in Málaga, Spain). Prior studies indicated [15–17] that at least 33% of these patients would not have an appropriate INR control level (INR values above 2 and under 3 or under 3.5 for prosthetic valve wearers). To reach an appropriate sample size in the estimated proportion, the following terms must be considered: (1) using a normal asymptotic confidence interval with a correction to finite populations on bilaterally of 95%, (2) assuming that the expected population is approximately 33%, and (3) since the population total size is 750, it was deemed necessary to include 312 experimental units in this study (Fig. 1).

#### Step II

Accepting an alfa risk of 0.05 and a beta risk of 0.20 in a bilateral comparison between the study group and the control group, 312 individuals will be required (156 in each group) to detect differences in INR figures equal or higher than 15% between the groups for the primary target. It has been estimated by the researches that there will be a tracking loss rate of 10%. It will be assumed these lost individuals are impossible to locate or are those who do not wish to take part or cannot attend the scheduled revisions, in accordance with the study intervention plan, as well as deaths occurring within 6 months of the intervention. Given the relevant impact of the development of cardioembolic or hemorrhagic events due to out of range INR control values, changes of approximately 15% could have significant clinical relevance.

Patients will be recruited from the list of patients taking anticoagulant drugs included in the digital medical record according to the inclusion and exclusion criteria. The recruitment period will last for 1 month.

To reach the required sample size, all patients from the four health centers on AOT using vitamin K antagonists will be studied. Patients will be selected from their own PCC once identified by “Seguridad Social de Andalucia” user number (NUSSA). Once the inappropriate individuals are identified, they will be consecutively placed considering their social security number in every health center. After that, patients in each strate (PCC) will be randomly distributed in the intervention group or the control group. The investigators will try to avoid bias depending on each health center issues.

It has been estimated a follow-up loss ratio of approximately 10%. Deaths 6 months from the intervention, and inability to locate individuals, or not attending to the scheduled meetings will be considered as losses when dealing with the study intervention program.

#### Study design

Randomized single-blind controlled trial.

### Description of the intervention

The study will be carried out over a 28-month period. It started on 1 September 2018, and it will finish on 31 December 2020. Participants will be contacted by phone. Trial information with details will be explained to them, and they will be asked whether they are interested in taking part. At the same time, they will be scheduled for the group meetings. Signed informed consent forms will be obtained when the patient attends the health center before the group meetings begin. Patients in each strate (PCC) will be randomly distributed to the intervention group or the control group (a random numbers table will be used). The allocation sequence will be generated by the researcher team. The allocation concealment mechanism consists of organizing patients considering their Andalusian Health Service Number of Clinical Record (AHNCR). After assignment to an intervention, participant names will be deleted by the nurses in charge of the recruitment in order to mask the data. The outcome assessors will not know the patients’ identity. The masking will be carried out by replacing the patients’ identification data with an assignment number.

All patients under study will receive an information sheet, and they will sign informed consent forms when they attend to INR control appointments. The investigators will try to encourage the involvement of every person so that patients included in the intervention group will be telephoned in previous weeks in order to achieve attendance at group meetings (at least three times and at different times if necessary) by a nurse. They will be recalled the day before the meeting as a reminder. After that, the intervention will be carried out and it will be based on the patients’ attendance of an educational session led by an expert patient (a member of the “Málaga anticoagulated patients association”). This person had been previously trained by health professionals and is able to take on a leadership role as an instructor, providing support through their knowledge and experience. Written material and a questionnaire form were developed for the intervention group before the meeting (Anexo VII). The patients are asked to answer questions about their vitamin K antagonist knowledge. Then, the expert patient will answer this question for everyone “what obstacles could we find when trying to reach an appropriate anticoagulation?” The instructor will rely on the support of short PowerPoint presentations as well as on graphic material explaining the subjects to the patients. The main explanations for inappropriate control values will be provided by the expert patient: other drug consumption, inadequate food, adherence to treatment, etc. Brainstorm, Phillips technique 6.6, role playing, and a table including main ideas provided by the patients will be used to assist them in keeping their INR levels in an appropriate range, and the impediments they face will be addressed. All of this will complement the knowledge transmitted by health professionals. A sanitary professional will carry out observations and energizer tasks if necessary.

Group meetings with educational sessions will be organized in a primary health center, and they will take place from Monday to Thursday in the evenings (according to the convenience of the center) with at least 90 min each. It has been estimated that eleven sessions are necessary, including 15 persons each as a maximum in order to complete one session per person. The sessions will all be held in the same classroom and under the management of the expert patient and the observation of the main researcher.

Every patient will be required to attend one group meeting (the intervention group). The control group will perform the usual clinical practice: people will be scheduled to visit with a nurse about once per month, except for cases in which controls are inappropriate who will be scheduled for more frequent visits. Patients in the control group will not attend any of the group sessions. Both groups will be followed with their usual INR controls, carrying out the needed number of measurements under the guidance of the health professionals involved in the appropriate INR control of all patients.

As the final step, once the intervention had been carried out for every indicated patient, INR figures and data will be collected over the next 6 months. The study participants will be able to withdraw their informed consent at any time, and they will be free to leave the study if they do not want to complete the 6 months of INR control after the educational intervention. The main reasons for discontinuing or modifying the interventions assigned to each study participant will be a withdrawal request, a participant’s death, or stopping the use of antivitamin K drugs. Unmasking will be allowed in those cases.

Protocol amendments will be reported to the research ethics committee and to the funding foundation (FIMABIS) and trial sponsor (FJNM).

#### Data collection

Plans to collect variables will consist of an analysis of the digital medical records in order to obtain the patients’ clinical information. Meetings have been carried before the beginning of the research among the researchers to unify data collecting criteria. Data collecting forms are available as attached documents to this protocol. All of the variables detailed below will be collected as long as they are suitably listed in the digital medical history or could be directly obtained from the patient during the medical interview, without the need for changing the health center’s usual clinical practice, over the 6 months prior to the intervention. Any data required to support the protocol can be supplied on request. The data will be collected on the collection sheets and kept by the principal investigator as follows.

#### First step

Patients on AOT programs will be enrolled from several primary health centers from a healthcare area of Málaga. They will be evaluated on their clinical characteristics. The measurements obtained from the digital clinical history over the last 6 months before the beginning of the study will be recorded, as well as the rest of the variables, in the collection sheets.

#### Second step

Once the study of the VKA anticoagulation control degree is performed, patients who present inappropriate control values will be selected. A list of patients with deficient INR control at the participant health centers will be made. An inappropriate INR control is defined as when the therapeutic INR values (range from 2 to 3, or to 3.5 in prosthetic valve wearers) percentages (TRT) are less than 65%, using the Rosendaal method to obtain them. In cases of no availability, INR controls will be considered inappropriate when the therapeutic INR value percentage results are less than 60% of the total measures. For any of the assumptions, the assessment period is at least the last 6 months. There is no anticipated harm and compensation for trial participation. As a provision for post-trial care, patients on inappropriate INR control figures will be indicated to stop vitamin K antagonist intake. They will be indicated to start a non-vitamin K antagonist oral anticoagulant (NOAC) intake if they suffer from atrial fibrillation and anticoagulation criteria exist.

The data monitoring committee is composed of the main researcher (LGM) and doctors ABO and RRG. This committee is independent of the sponsor, and funding is not made under any term.

### Variables: operative definition

The main variable to be assessed in this study is time on INR therapeutic levels in the last 6 months of receiving VKA treatment. Appropriate INR level control will be assessed in two ways: measuring the therapeutic INR value percentages or time percentages in therapeutic values estimated using the Rosendaal method. It records the figure percentages as defined by their acceptable values according to the pathology the patient suffers from. This is from 2 to 3 for atrial fibrillation or from 2.5 to 3.5 if thromboembolism prevention is carried out after a heart valve surgery has been performed. Therefore, this quantitative variable had to be measured to calculate the patient’s percentage of an appropriate range, according to the patient’s condition.

Independent variables, also called study variables, are those that could affect the result variables. We classified them into eight groups to make their analysis easier.

Sociodemographic: age and sex, marital status, employment situation, and social and family support.

Somatometry and vital signs: height, weight, body mass index (BMI), diastolic and systolic blood pressure, and heart rate.

Cardiovascular risk factors: smoking habit, diabetes, arterial hypertension, dyslipidemia, and chronic renal failure.

Cardiovascular conditions: atrial fibrillation (AF), interventional cardiac valve diseases with or without AF, ischemic cardiopathy, thromboembolic disease history (stroke, transient ischemic attack), hemorrhagic stroke, and congestive heart failure.

Blood test: complete blood count, bleeding time and prothrombin time or partial thromboplastin time, glomerular filtration rates, total cholesterol, HDL and LDL cholesterol, and triglycerides. They were obtained from the last blood test carried out for the patient.

Use of accompanying medication: total number of drugs and anticoagulant type.

Consumption of “gastric protector” or nonsteroidal anti-inflammatories.

Dietary habits: usual consumption of food rich in vitamin K, alcohol consumption, and frequent diet transgressions.

### Primary outcome measure

1. Time on INR therapeutic levels in the last 6 months of receiving VKA treatment: Appropriate INR level controls will be assessed in two ways: measuring the therapeutic INR value percentages or measuring time percentages in therapeutic values estimated using the Rosendaal method [time frame, 0 and 6 months]

### Secondary outcome measures

2. Sociodemographic: record of age and sex [time frame, start of the study]
3. Body mass index (BMI): determination of the body mass index calculated as the weight measured in kilograms (kg) divided by the height measured in meters squared (weight/height^2^) (kg/m^2^) [time frame, 0 and 6 months]
4. Diastolic and systolic blood pressure: determination of diastolic and systolic blood pressure measured in millimeters of mercury (mm/Hg), average of 2 determinations [time frame, 0 and 6 months]
5. Heart rate: determination of the number of heartbeats per minute, average of 2 determinations [time frame, 0 and 6 months]
6. Smoking habit: incidence of smoker’s habit status in the electronic medical record [time frame, 0 and 6 months]
7. Diabetes: incidence of the condition of diabetes in the electronic medical record [time frame, 0 and 6 months]
8. Arterial hypertension: incidence of the diagnosis of hypertension in the electronic medical record [time frame, 0 and 6 months]
9. Dyslipidemia: incidence of the diagnosis of dyslipidemia in the electronic medical record [time frame, 0 and 6 months]
10. Chronic renal failure: incidence of the diagnosis of chronic renal failure in the electronic medical record [time frame, 0 and 6 months]
11. Atrial fibrillation: incidence of the diagnosis of atrial fibrillation in the electronic medical record [time frame, 0 and 6 months]
12. Interventional cardiac valve diseases with or without atrial fibrillation: incidence of the diagnosis of interventional cardiac valve diseases in the electronic medical record [time frame, 0 and 6 months]
13. Ischemic cardiopathy: incidence of the diagnosis of ischemic cardiopathy in the electronic medical record [time frame, 0 and 6 months]
14. Thromboembolic disease history (stroke, transient ischemic attack): incidence of a thromboembolic disease history (stroke, transient ischemic attack) in the electronic medical record [time frame, 0 and 6 months]
15. Hemorrhagic stroke: incidence of the diagnosis of hemorrhagic stroke in the electronic medical records [time frame, 0 and 6 months]
16. Congestive heart failure: incidence of the diagnosis of congestive heart failure in the electronic medical records [time frame, 0 and 6 months]
17. Glomerular filtration rates: determination of the total glomerular filtration rates in ml/min/1.73 m^2^; change from basal to 12 months [time frame, 0 and 6 months]
18. Total cholesterol: determination of total cholesterol measured in milligrams per deciliter (mg/dl); change from basal to 12 months [time frame, − 6 and 6 months]
19. HDL cholesterol: determination of high-density lipoprotein cholesterol (HDLc); LDLc measured in milligrams per deciliter (mg/dl) (low-density lipoprotein cholesterol); HDLc measured in milligrams per deciliter (mg/dl); change from basal to 6 months [time frame, − 6 and 6 months]
20. LDL cholesterol: determination of low-density lipoprotein cholesterol (LDLc); LDLc measured in milligrams per deciliter (mg/dl); change from basal to 6 months [time frame − 6 and 6 months]
21. Triglycerides: determination of total triglycerides measured in milligrams per deciliter (mg/dl); change from basal to 12 months [time frame, − 6 and 6 months]
22. Total number of drugs: determination of the total number of different medications prescribed in the electronic medical records; change from basal to 12 months [time frame, − 6 and 6 months]
23. Anticoagulant type: determination of the anticoagulant type prescribed in the electronic medical records; change at baseline and at 12 months [time frame, − 6 and 6 months]
24. Consumption of “gastric protector”: determination of the consumption of “gastric protector” prescribed in the electronic medical records; change at baseline and at 12 months [time frame − 6 and 6 months]
25. Usual consumption of food rich in vitamin K: change in usual consumption of food rich in vitamin K; change at baseline at 12 months [time frame, − 6 and 6 months]
26. Alcohol consumption: change in usual alcohol consumption; change at baseline and at 12 months [time frame, − 6 and 6 months]
27. Eligibility: minimum age, 18 years; sex, all; accepts healthy volunteers, no

### Statistical analysis

First of all, a descriptive analysis of the study variables will be carried out. Continuous variable values will be summed in an index which will show the means and standard deviation or medians according to the variable distribution (symmetric or asymmetric, respectively) and the values range: maximum and minimum. Categorical variables will be presented using absolute and relative frequencies. Chi-square test will be applied to analyze the observed differences in qualitative variable frequencies or Fisher’s test if under 5, weighted value percentage amounts are more than 20%. Odds ratio and its confidence intervals at 95% will be calculated in the case of two-dimensional indexes. Student’s *t* test for paired samples will be applied to study the differences in continuous variables, before and after the intervention, in cases that the normality requirement could be accepted, after applying the Shapiro-Wilk test. To compare the obtained values from the experimental group to the control group, Student’s *t* test will be used for independent groups or its non-parametrical equivalent, the Mann-Whitney test. If normality cannot be accepted, non-parametrical Wilcoxon test should not be applied. In cases of obtaining significantly statistical results, confidence intervals at 95% will be calculated. Multiple linear regression models will be used to determine the factors associated with inappropriate INR control. An intention-to-treat (ITT) analysis will be used.

Statistical analysis will be conducted using R software 3.0 version (Foundation for Statistical Computing, Vienna, Austria; available at http://www.R-project.org 1.5.11).

### Risks or limitations and viability

Some of the studied variables may not be collected in the digital clinical history, which is the main data source. To solve this limitation and considering that patients have to attend necessary to INR controls in the health center, the missing data will be recovered using a clinical interview. Furthermore, we could experience low group meeting attendance due to lack of interest or lack of motivation of the studied individuals. Patients who do not attend a meeting will be called again to participate in the next group session. “Hawthorne effect” could occur namely when patients feel that they are being observed, it forces them to comply with the advices in a more strict way. Furthermore, the main investigator will attend the intervention group meetings, which may result in bias when he interprets the outcomes.

The research will be carried out under usual clinical practice. The intervention that will be performed is strictly educative. Potential side effects are associated with patient risks linked to anticoagulant drug intake.

### Timetable and work program

The study is being carried out over a 28-month period. The assembly of the project took place on 1 day at the Primary Care Center *Ciudad Jardin* practice classroom for all healthcare members involved in the research, along with the expert patient who will execute the intervention. The nurses will explain to the participants about the study approach, and they will collect the consent forms from the participants.

In the first step, at the start of the study, a retrospective data collection of the 6 months prior to the intervention will be completed from all patients who meet the selection requirements and are included in the AOT program. Once the anticoagulated patients have been analyzed and the list of the patients on inappropriate control has been completed, a random sampling will be done in every health center in order to build two groups: *intervention group and control group*.

The intervention group will include the indicated patients. Nurse students will take on the telephone calls to schedule intervention group patients for group meeting attendance. The main researcher and the expert patient will attend every group meeting, but the researcher will act just as an observer. Every participant center will rely on nurses (10 partners) and two resident medical interns (learning Family and Community Medicine) to complete this task. Due to their frequent contacts with anticoagulated patients, the nurses will instruct them about study issues and will collect the signed written consent forms; also, they will be involved in data collecting, INR figures, and patient study variables.

A portfolio with the collected data forms will be distributed to every participant. It will be employed by the patients to collect their data, which will be transcribed to a digital book. This book will be identical for every participant center and it will be used as a basis for the randomization. Two resident medical interns will handle all of the computerized recordings.

**Fig. 2:**
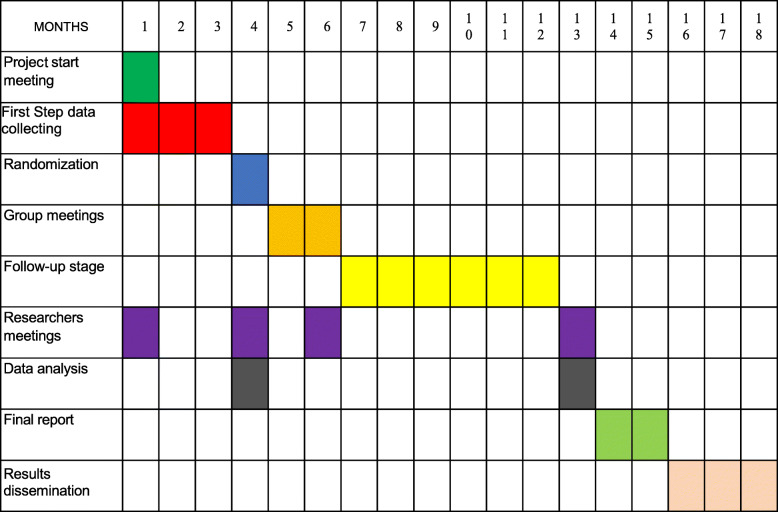
Timetable

After that, the intervention will be carried out and it will be based on the patients’ attendance of one meeting lead by an expert patient. Due to the lack of space and in order to improve the patient understanding, and according to the convenience of the center, the meeting will be performed on different evenings but always in the same classroom and under the management of the expert patient and the observation of the main researcher. The last step will consist of collecting INR control data. INR control data will be obtained within 6 months from the activity date in both groups (control group and the intervention group). Then, a new assembly of the researchers’ team will be carried out. Once this period expires, a comparative before-after statistical study of every studied subject and between the intervention and control group will be performed (Fig. 2).

An analysis to check the proper operation of the intervention will be made following the first group meeting.

The data monitoring committee is comprised of the main researcher (LGM) and doctors ABO and RRG. All researchers will have access to the final data set. The results will be reported to the participants within 6 months after the statistical analysis is completed. This committee is independent of the sponsor, and funding is not provided under any terms or competing interests.

### Legal and ethical issues

This project will be performed respecting the principles of the Declaration of Helsinki (Fortaleza Revision. 2013) and Good Clinical Practice. Personal data will be treated following European Parliament Resolution 2016/679 and 27th of April of 2016 Council with concerns of personal data treatment and protection.

The study protocol was approved by the research ethical committee which belongs to the General Healthcare Area of Málaga, prior to initiation of patient recruitment on 26 March 2015.

Data confidentiality is always an imperative condition, and the data use was strictly conducted to reach the protocolized aims and to notify the relevant authorities.

Protocol amendments will be reported to the research ethics committee and to funding foundation (FIMABIS) and trial sponsor (FJNM).

## Data Availability

Available upon justified request to the author.

## Abbreviations

DAOA: Direct action oral anticoagulants
VKA: Vitamin K antagonists
HC: Health center
AF: Atrial fibrillation
AH: Arterial hypertension
BMI: Body mass index
INR: International normalized ratio
AOT: Oral anticoagulant therapy
TRT: Time in therapeutic range
